# Development and validation of a 24-h predictive model for hypertriglyceridemic moderately severe acute pancreatitis: a single-center retrospective study

**DOI:** 10.3389/fmed.2026.1862797

**Published:** 2026-07-02

**Authors:** Songlong Yang, Chaoqun Li, Kaiping Zeng, Minguang Zhang, Wenrong Wang

**Affiliations:** 1 Department of Spleen-Stomach-Hepatobiliary, Quanzhou Hospital of Traditional Chinese Medicine Affiliated to Fujian University of Traditional Chinese Medicine, Quanzhou, Fujian, China; 2 Department of Spleen-Stomach-Hepatobiliary, Quanzhou Hospital of Traditional Chinese Medicine, Quanzhou, Fujian, China; 3 Department of Gastroenterology, The Second People's Hospital Affiliated to Fujian University of Traditional Chinese Medicine, Fuzhou, Fujian, China; 4 Fujian Clinical Medical Research Centre of Chinese Medicine for Spleen and Stomach, The Second People's Hospital Affiliated to Fujian University of Traditional Chinese Medicine, Fuzhou, Fujian, China

**Keywords:** early prediction, hypertriglyceridemia, moderately severe acute pancreatitis, predictive model, SIRS

## Abstract

**Objective:**

To develop and validate a risk prediction model for identifying patients with hypertriglyceridemia-induced acute pancreatitis who are at risk of progressing to moderately severe acute pancreatitis (MSAP) within 24 h after admission, thereby providing decision support for early clinical intervention.

**Methods:**

A single-center retrospective study was conducted, enrolling 146 patients with HTG-AP admitted between January 2018 and December 2023. The patients were randomly divided into a training set and a validation set at a ratio of 7:3. Predictive factors were screened using univariate analysis and multivariate logistic regression, To address the quasi-complete separation observed in the SIRS variables, a Firth penalized likelihood logistic regression model was employed to develop the prediction model. Model discrimination was assessed using the area under the receiver operating characteristic curve (ROC-AUC). Calibration performance was evaluated using the Hosmer–Lemeshow goodness-of-fit test, calibration plots, Brier score, and calibration slope. Clinical utility was assessed through decision curve analysis (DCA). Comparative performance against the BISAP score was conducted using DeLong’s test. Internal validation was performed using 1,000 bootstrap resamples to estimate and adjust for optimism bias.

**Results:**

Multivariable analysis based on Firth’s penalized likelihood logistic regression showed that systemic inflammatory response syndrome (SIRS) (OR = 202.469, *p* < 0.001) and elevated triglyceride (TG) levels (OR = 1.066, *p* = 0.013) within 24 h after admission were independent risk factors for predicting moderately severe disease. In the validation cohort, the area under the ROC curve (AUC) of the prediction model was 0.909, with a sensitivity of 86.2% and a specificity of 90.9%. In comparison, the AUC of the 24-h TG level alone (cut-off value: 18.41 mmol/L) was 0.690, with a sensitivity of 58.62% and a specificity of 81.82%.

**Conclusion:**

SIRS and elevated TG levels within 24 h after admission are independent predictors of progression to moderately severe disease in patients with HTG-AP. The prediction model based on these variables demonstrates good predictive performance. A 24-h TG level below 18.41 mmol/L indicates a relatively lower risk of progression to moderately severe disease; however, prediction based on combined biochemical indicators is recommended. Further studies are required to validate the effectiveness of this model.

## 1 Introduction

Hypertriglyceridemic acute pancreatitis (HTG-AP) is an important subtype of acute pancreatitis (AP), and its incidence has been increasing worldwide. It has become the third most common cause of AP after gallstones and alcohol (1, 2). In China, HTG-AP has risen to the second leading cause, accounting for more than 25% of all AP cases (3, 4). Compared with AP caused by other etiologies, patients with HTG-AP often present with more severe clinical manifestations, including a higher risk of organ failure, an increased incidence of local complications, longer hospital stays, and poorer clinical outcomes (5–7).

Since the revised Atlanta classification was introduced in 2012, numerous studies have focused on identifying risk predictors and developing predictive models for severe acute pancreatitis (SAP). However, relatively few studies have examined predictors of moderately severe acute pancreatitis (MSAP), and early identification of this condition before 48 h remains challenging (8). A key distinction between moderately severe and severe disease is whether organ failure resolves within 48 h. Therefore, early identification of patients at high risk of progressing to moderately severe disease within the first 24 h after admission, followed by timely clinical intervention, may effectively reduce the likelihood of progression to severe disease and improve patient outcomes.

Currently, the clinical assessment of AP severity mainly relies on the Atlanta classification and several established scoring systems. These evaluation tools typically incorporate clinical manifestations, imaging findings, or complex laboratory parameters, including the Ranson score, BISAP score, APACHE II score, and CTSI score, which are usually assessed over a period of 48–72 h. Although these scoring systems have demonstrated good accuracy in evaluating disease severity and predicting prognosis, they have certain limitations, such as delayed predictive timing, relatively limited efficiency for early prediction, or the requirement for complex parameter calculations. These limitations may hinder timely identification of high-risk patients and delay early therapeutic intervention (9–11). Previous studies have also explored several biochemical markers, such as C-reactive protein (CRP) (12) and Ca^2+^ (13), for predicting AP severity; however, these markers typically reach peak levels only 48–72 h after disease onset. Consequently, many centers have attempted to analyze their own clinical datasets to develop early predictive models capable of more accurately identifying disease progression (14).

Based on this background, the present study was designed as a single-center retrospective cohort analysis to develop a simplified prediction tool using easily accessible clinical indicators obtained within 24 h after admission. The aim was to address the current gap in rapid risk stratification for patients with hypertriglyceridemic moderately severe acute pancreatitis (HTG-MSAP). By collecting clinical and laboratory data from patients with HTG-AP at 24 h and 72 h after admission, a predictive model was constructed and subsequently validated using statistical methods.

## 2 Materials and methods

### 2.1 Subjects

The clinical data of 172 patients diagnosed with mild or moderately severe HTG-AP who were admitted to the Department of Spleen-Stomach-Hepatobiliary, Quanzhou Hospital of Traditional Chinese Medicine between January 2018 and December 2023 were retrospectively analyzed. The clinical data for this study were accessed from the hospital electronic medical record system on 02/03/2026. After applying the predefined inclusion and exclusion criteria, a total of 146 patients were ultimately included in the study. This study was conducted as a retrospective analysis and was approved by the Ethics Committee of Quanzhou Hospital of Traditional Chinese Medicine ([2026] NO.002). The requirement for informed consent was waived due to the retrospective nature of the study.

### 2.2 Main definitions and diagnostic criteria

Diagnostic criteria for AP: According to the revised Atlanta Classification published in 2012 (15), the diagnosis of AP requires at least two of the following three criteria: Characteristic abdominal pain consistent with AP (severe, acute, and persistent upper abdominal pain often radiating to the back). Serum amylase and/or lipase levels at least three times higher than the upper limit of normal. Imaging findings showing typical features of AP. Diagnostic criteria for HTG-AP: HTG-AP was diagnosed when AP was confirmed and the serum triglyceride (TG) level at the onset of symptoms was ≥11.3 mmol/L (1,000 mg/dL), or between 5.65 and 11.3 mmol/L (500–1,000 mg/dL) in the absence of other etiologies such as biliary disease or alcohol consumption (16).

Severity classification of AP: The severity of AP was classified according to the 2012 revised Atlanta Classification (15). Mild acute pancreatitis (MAP) was defined as AP without organ failure or local or systemic complications. MSAP was defined as the presence of transient organ failure (resolving within 48 h) and/or local or systemic complications. SAP was defined as persistent organ failure (single or multiple organs) lasting longer than 48 h.

Diagnostic criteria for systemic inflammatory response syndrome (SIRS): SIRS was diagnosed according to the consensus criteria established by the American College of Chest Physicians (ACCP) and the Society of Critical Care Medicine (SCCM) in 1992 (17). The diagnostic criteria include the following: Abnormal body temperature: >38 °C or <36 °C. Tachycardia: heart rate >90 beats/min. Tachypnea: respiratory rate >20 breaths/min or arterial partial pressure of carbon dioxide (PaCO_2_) < 32 mmHg. Abnormal white blood cell count (WBC): >12 × 10^9^/L or <4 × 10^9^/L, immature granulocytes. SIRS was diagnosed when two or more of the above criteria were present.

Bedside Index for Severity in Acute Pancreatitis (BISAP) scoring criteria (18): blood urea nitrogen (BUN) > 25 mg/dL (8.9 mmol/L), impaired mental status, SIRS, age > 60 years, and pleural effusion; each item is assigned 1 point. A total score of ≥3 indicates high risk.

### 2.3 Inclusion and exclusion criteria

#### 2.3.1 Inclusion criteria

1. Diagnosis of mild or moderate severe HTG-AP.2. Admission to hospital within 24 h after the onset of symptoms. 3. Be over 18-year-old.4. Hospital stay longer than 72 h. 5. Availability of laboratory obtained within 24 h after admission.

#### 2.3.2 Exclusion criteria

1. No laboratory tests performed within 24 h after admission. 2. Hospital stay shorter than 72 h. 3. Age under 18 years.4. Missing clinical data exceeding 20%.

### 2.4 Treatments

Early treatment measures included fasting, inhibition of pancreatic enzyme secretion, suppression of gastric acid secretion, and fluid resuscitation. Lipid-lowering therapy was administered using low-molecular-weight heparin combined with insulin. Analgesic therapy was provided when necessary. In addition, conventional traditional Chinese medicine (TCM) treatment, including oral administration and enema, was applied. Antibiotics were administered when infection was clinically suspected or confirmed.

### 2.5 Observation indicators

(1) General characteristics: sex, age, and history of alcohol records.
(2) Clinical characteristics: disease severity, presence of diabetes mellitus, presence of diabetic ketosis or ketoacidosis, and the occurrence of SIRS.
(3) Laboratory tests: WBC, Platelet Count (PLT), Hematocrit (HCT), Mean Platelet Volume (MPV), Platelet volume Distribution Width (PDW), Red Blood Cell Count (RBC), Hemoglobin (HGB), CRP, Triacylglycerol (TG), Urea Nitrogen (UN), Serum Creatinine (SCr), Calcium (Ca^2+^) at 24 h and 72 h after admission.

### 2.6 Research and statistical methods

According to disease severity, patients were divided into a mild group and a moderately severe group. A stratified random sampling method was applied to divide the dataset into two subsets: a training set (106 cases) and a validation set (40 cases), corresponding to 70 and 30% of the total sample, respectively. This approach ensured that the outcome distribution in the validation set was consistent with that of the training set.

For continuous variables with a normal distribution, data were expressed as the mean ± standard deviation (Mean ± SD), and comparisons between groups were performed using the independent-samples *t* test. Continuous variables with a non-normal distribution were expressed as the median and interquartile range [M (Q1, Q3)], and comparisons between groups were conducted using the Wilcoxon rank-sum test. Categorical variables were presented as frequency and percentage [*n* (%)], and comparisons between groups were performed using the chi-square test. Variables with *p* < 0.05 in the univariate analysis were considered statistically significant and were subsequently included in the multivariate analysis. Because the SIRS variable exhibited quasi-complete separation in this study, and to mitigate the bias of maximum likelihood estimation caused by the small sample size and separation, the final prediction model was developed using Firth’s penalized likelihood logistic regression, with moderate-to-severe disease as the positive outcome. Model discrimination was assessed using the receiver operating characteristic (ROC) curve and area under the curve (AUC). Calibration performance was comprehensively evaluated using the Hosmer–Lemeshow goodness-of-fit test, calibration curve, Brier score, and calibration slope. Clinical utility was assessed using DCA. The performance of the final model was further compared with the clinically commonly used BISAP score using ROC analysis and DeLong’s test. Given the absence of an external validation cohort, internal validation was performed using 1,000 bootstrap resampling iterations to estimate optimism bias and improve the robustness of the performance estimates.

An independent internal validation set (*n* = 40) was used to evaluate the final Firth-based model. The regression equation derived from the training set was fixed, and predicted probabilities were calculated for each patient in the validation cohort. Based on these probabilities, ROC curves were reconstructed, and the AUC, sensitivity, specificity, accuracy, and optimal cutoff value were determined. Calibration was also assessed using the same predicted probabilities, including the Hosmer–Lemeshow test, calibration curve, Brier score, and calibration slope.

To further evaluate the clinical value of the 24-h TG level as a single predictor of disease severity, a single-variable ROC curve analysis was performed in the training set. The AUC and its 95% confidence interval (CI) were calculated, and the optimal cut-off value was determined by maximizing the Youden index. Sensitivity, specificity, positive predictive value (PPV), negative predictive value (NPV), and accuracy were then calculated in the validation set to evaluate its predictive performance.

All statistical analyses were performed using SAS software (version 9.4). A two-sided *p* < 0.05 was considered statistically significant.

## 3 Results

### 3.1 Basic information of the dataset

#### 3.1.1 Comparison between training set and validation set

According to the study design, the samples were divided into a training set (*n* = 106) and a validation set (*n* = 40) at a ratio of 70% for the training set and 30% for the internal validation set. The baseline characteristics and laboratory parameters of the two datasets were compared. The results showed that there were no statistically significant differences in most baseline characteristics or laboratory indicators between the two datasets (*p* > 0.05). The only exceptions were the 24-h WBC level, which was slightly higher in the validation set than in the training set (15.33 vs. 13.79, *p* = 0.048), and the 72-h blood UN level, which was slightly higher in the training set than in the validation set (4.5 vs. 4.1, *p* = 0.035). Considering the overall balance of the remaining variables, these minor differences in individual indicators were unlikely to affect the overall comparability of the two groups. Therefore, the two datasets were considered comparable and suitable for subsequent model training and validation. The detailed results are presented in Table 1.

**Table 1:** Comparison of baseline data and laboratory indicators between training set and validation set (*n* = 146).

Variables	Training set (*n* = 106)	Validation set (*n* = 40)	χ^2^/*t*/*Z*	*p* value
Gender (M/FM)	92/14	33/7	0.4345	0.510
Age (Y)	41.97 ± 8.25	39.63 ± 8.17	1.54	0.127
Drinking (Y)	67 (64.4%)	20 (50.0%)	2.5129	0.113
Severity (MAP/MSAP)	71 (66.98%)	29 (72.50%)	0.4099	0.522
Diabetes (Y)	65 (61.32%)	24 (60.00%)	0.0213	0.884
Ketosis (Y)	43 (40.95%)	17 (42.50%)	0.0286	0.866
Acidosis (Y)	12 (11.32%)	5 (12.50%)	0.0393	0.843
SIRS (Y)	69 (65.71%)	26 (65.00%)	0.0065	0.936
24-h indicator				
WBC × 10^9^/L	13.79 ± 4.07	15.33 ± 4.40	−1.99	0.048
CRP (mg/L)	26.4 (7.9,70.4)	25.9 (7.0,68.8)	−0.131	0.896
PLT × 10^9^/L	236.5 (190.0,285.0)	249.0 (210.5,314.0)	1.483	0.138
HCT (%)	0.435 ± 0.041	0.433 ± 0.045	0.32	0.751
MPV (fL)	9.7 (8.4,10.3)	9.8 (9.2,10.3)	0.529	0.597
PDW (%)	52.68 ± 8.36	50.58 ± 8.12	1.37	0.173
RBC × 10^12^/L	5.13 ± 0.55	5.04 ± 0.53	0.91	0.364
HGB (g/L)	159.2 ± 22.59	158.8 ± 17.07	0.09	0.928
TG (mmol/L)	21.5 (12.2,39.3)	16.4 (9.7,27.8)	−1.76	0.078
UN (mmol/L)	4.5 (3.6,5.4)	4.1 (2.8,4.9)	−2.111	0.035
SCr (μmol/L)	77.46 ± 25.00	73.00 ± 18.48	1.03	0.307
CA^2+^ (mmol/L)	2.7 (2.1,2.4)	2.2 (2.2,2.4)	0.604	0.546
72-h indicator				
WBC × 10^9^/L	9.36 ± 3.45	9.67 ± 4.05	−0.45	0.651
CRP (mg/L)	131.0 (49.0,207.0)	122.0 (65.8,194.0)	−0.062	0.950
PLT × 10^9^/L	193 (160,234)	210.5 (167.0,254.5)	1.268	0.205
HCT (%)	0.388 ± 0.046	0.379 ± 0.048	1.04	0.301
MPV (fL)	10.0 (9.1,10.9)	9.9 (9.5,10.4)	−0.051	0.960
PDW (%)	50.45 ± 7.01	49.74 ± 8.85	0.51	0.612
RBC × 10^12^/L	4.49 ± 0.64	4.36 ± 0.57	1.17	0.245
HGB (g/L)	132.3 ± 15.82	130.8 ± 18.22	0.49	0.624
TG (mmol/L)	4.7 (3.4,7.7)	4.9 (3.4,6.9)	−0.11	0.913
UN (mmol/L)	3.5 (2.6,4.1)	3.5 (2.5,4.5)	0.417	0.676
SCr (μmol/L)	68.64 ± 15.88	63.56 ± 17.16	1.55	0.123
CA^2+^ (mmol/L)	2.1 (2.0,2.2)	2.2 (2.1,2.3)	1.628	0.104

#### 3.1.2 Training set general information

A total of 106 patients were included in the training set, comprising 35 cases in the mild group and 71 cases in the moderately severe group. There were no significant differences between the two groups in terms of sex distribution, age, alcohol consumption history, or laboratory indicators at 24 h, including WBC, CRP, and PLT (all *p* > 0.05).

The incidence of comorbidities was significantly higher in the moderately severe group than in the mild group, including diabetes mellitus (69.0% vs. 45.7%), ketosis (47.9% vs. 26.5%), acidosis (16.9% vs. 0%), and SIRS (94.3% vs. 8.6%) (all *p* < 0.05).

Regarding laboratory indicators, the 24-h TG level in the moderately severe group was significantly higher than that in the mild group [27.0 (13.15, 43.79) mmol/L vs. 15.2 (8.9, 28.4) mmol/L, *Z* = −3.164, *p* = 0.002]; At 72 h, WBC [9.9 ± 3.4 × 10^9^/L vs. 8.1 ± 3.3 × 10^9^/L, *t* = −2.62, *p* = 0.01], CRP level [167.0 (86.3,224.0) mg/L vs. 47.1 (23.6,127.0) mg/L, *Z* = −4.171, *p* < 0.001] were significantly higher in the moderately severe group, whereas HCT [0.381 ± 0.050% vs. 0.402 ± 0.032%, *t* = 2.29, *p* < 0.001; *p* = 0.024], HGB [129.8 ± 17.1 g/L vs. 137.5 ± 11.4 g/L, *t* = 2.42, *p* = 0.017], and CA^2+^ [2.1 (2.0,2.2) mmol/L vs. 2.2 (2.1,2.3) mmol/L, *t* = 2.42, *p* = 0.017]. *Z* = 4.190, *p* < 0.001 were significantly lower than those in the mild group, and the differences were statistically significant. The detailed results are presented in Table 2.

**Table 2:** Comparison of baseline data and laboratory indicators between the two groups in the training set (*n* = 106).

Variables	MAP (*n* = 35)	MSAP (*n* = 71)	χ^2^/*t*/*Z*	*p* value
Gender (M/FM)	31/4	61/10	0.144	0.704
Age (Y)	43.5 ± 6.4	41.2 ± 9.0	1.52	0.133
Drinking (Y)	23 (65.7%)	44 (63.8%)	0.038	0.845
Diabetes (Y)	16 (45.7%)	49 (69.0%)	5.366	0.021
Ketosis (Y)	9 (26.5%)^5^	34 (47.9%)	4.361	0.037
Acidosis (Y)	0 (0%)	12 (16.9%)	6.671	0.010
SIRS (Y)	3 (8.6%)	66 (94.3%)	76.087	<0.001
24-h indicator				
WBC × 10^9^/L	12.8 ± 3.3	14.3 ± 4.3	−1.84	0.069
CRP (mg/L)	10.6 (5.94,58.05)	28.2 (10.4,81.3)	−1.763	0.081
PLT × 10^9^/L	237 (188,274)	236 (190,289)	−0.205	0.838
HCT (%)	0.437 ± 0.029	0.434 ± 0.045	0.42	0.676
MPV (fL)	9.8 (8.9,10.3)	9.7 (8.4,10.5)	0.673	0.502
PDW (%)	52.02 ± 7.41	53.01 ± 8.82	−0.57	0.567
RBC × 10^12^/L	5.10 ± 0.47	5.14 ± 0.59	−0.36	0.718
HGB (g/L)	153.4 ± 29.6	162.0 ± 17.8	−1.85	0.067
TG (mmol/L)	15.2 (8.9,28.4)	27.0 (13.15,43.79)	−3.164	0.002
UN (mmol/L)	4.8 (3.6,5.7)	4.2 (3.5,5.3)	1.622	0.105
SCr (μmol/L)	81.2 ± 25.0	75.6 ± 25.0	1.09	0.277
CA^2+^ (mmol/L)	2.3 (2.2,2.4)	2.2 (2.1,2.3)	1.926	0.057
72-h indicator				
WBC × 10^9^/L	8.1 ± 3.3	9.9 ± 3.4	−2.62	0.01
CRP (mg/L)	47.1 (23.6,127.0)	167.0 (86.3,224.0)	−4.171	<0.001
PLT × 10^9^/L	207 (182,249)	191 (145,228)	1.787	0.077
HCT (%)	0.402 ± 0.032	0.381 ± 0.050	2.29	0.024
MPV (fL)	10.0 (9.3,11.6)	9.9 (9.0,10.7)	0.938	0.351
PDW (%)	48.69 ± 7.11	51.32 ± 6.85	−1.84	0.069
RBC × 10^12^/L	4.63 ± 0.44	4.42 ± 0.71	1.55	0.125
HGB (g/L)	137.5 ± 11.4	129.8 ± 17.1	2.42	0.017
TG (mmol/L)	5.02 (3.99, 7.60)	4.42 (3.12, 7.75)	0.637	0.524
UN (mmol/L)	3.45 (2.85, 4.10)	3.45 (2.50, 4.10)	0.035	0.972
SCr (μmol/L)	73.1 ± 16.3	66.6 ± 15.4	1.83	0.07
CA^2+^ (mmol/L)	2.2 (2.1,2.3)	2.1 (2.0,2.2)	4.190	<0.001

#### 3.1.3 Validation set general information

A total of 40 patients were included in the validation set, comprising 11 cases in the mild group and 29 cases in the moderately severe group. There were no significant differences between the two groups in age, sex, alcohol consumption history, diabetes history, or the incidence of ketoacidosis and acidosis (*p* > 0.05). However, the incidence of SIRS was significantly higher in the moderately severe group than in the mild group (86.2% vs. 9.1%, *p* < 0.001).

Regarding laboratory indicators, there were no significant differences between the two groups within 24 h after admission (*p* > 0.05). At 72 h after admission, WBC and CRP levels were significantly higher in the moderately severe group than in the mild group (*p* < 0.05), whereas CA levels were significantly lower (*p* < 0.05). No significant differences were observed between the groups in PLT, HCT, MPV, PDW, RBC, HGB, TG, UN, or SCr at 72 h (*p* > 0.05). Detailed data are presented in Table 3.

**Table 3:** Comparison of baseline data and laboratory indicators between the two groups of patients in the validation set (*n* = 40).

Variables	MAP (*n* = 11)	MSAP (*n* = 29)	χ^2^/*t*/*Z*	*p* value
Gender (M/FM)	9/2	24/5	-	1.000*
Age (Y)	40.4 ± 9.0	39.3 ± 8.0	0.35	0.730
Drinking (Y)	5 (45.5%)	15 (51.7%)	0.125	0.723
Diabetes (Y)	5 (45.5%)	19 (65.5%)	-	0.295*
Ketosis (Y)	3 (27.3%)	14 (48.3%)	-	0.297*
Acidosis (Y)	2 (18.2%)	3 (10.3%)	-	0.603*
SIRS (Y)	1 (9.1%)	25 (86.2%)	-	<0.001*
24-h indicator				
WBC × 10^9^/L	14.41 ± 2.50	15.68 ± 4.93	−0.81	0.423
CRP (mg/L)	9.2 (6.2,42.0)	32.3 (12.1,72.5)	−1.511	0.131
PLT × 10^9^/L	245 (225,276)	250 (208,329)	−0.182	0.856
HCT (%)	0.437 ± 0.029	0.434 ± 0.045	0.42	0.676
MPV (fL)	9.2 (8.2,10.0)	10.0 (9.2,10.3)	−1.958	0.05
PDW (%)	50.26 ± 4.14	50.69 ± 9.25	−0.15	0.884
RBC × 10^12^/L	5.11 ± 0.40	5.01 ± 0.57	0.54	0.591
HGB (g/L)	157.3 ± 9.55	159.4 ± 19.29	−0.34	0.732
TG (mmol/L)	12.1 (9.7,18.3)	19.8 (11.7,36.5)	−1.363	0.173
UN (mmol/L)	4.2 (3.5,5.5)	3.6 (2.7,4.7)	1.334	0.182
SCr (μmol/L)	80.00 ± 12.11	70.34 ± 19.91	1.5	0.142
CA^2+^ (mmol/L)	2.3 (2.2,2.3)	2.2 (2.2,2.4)	−0.387	0.699
72-h indicator				
WBC × 10^9^/L	7.84 ± 1.57	10.37 ± 4.49	−2.64	0.012
CRP (mg/L)	48.2 (7.8,75.9)	168.5 (104.0,228.0)	−3.127	0.002
PLT × 10^9^/L	201 (169,253)	214 (167,259)	−0.333	0.739
HCT (%)	0.384 ± 0.029	0.377 ± 0.054	0.43	0.673
MPV (fL)	9.9 (8.9,10.1)	9.9 (9.5,10.4)	−1.32	0.187
PDW (%)	48.13 ± 4.06	50.36 ± 10.09	−0.71	0.484
RBC × 10^12^/L	4.50 ± 0.34	4.30 ± 0.63	0.99	0.327
HGB (g/L)	135.5 ± 10.96	129.0 ± 20.19	1.01	0.319
TG (mmol/L)	4.64 (2.43,5.64)	4.98 (3.41,8.45)	−0.813	0.416
UN (mmol/L)	3.7 (3.3,4.5)	3.3 (2.3,4.5)	1.097	0.273
SCr (μmol/L)	70.40 ± 15.27	60.71 ± 17.40	1.53	0.136
CA^2+^ (mmol/L)	2.3 (2.2,2.4)	2.1 (2.0,2.2)	2.057	0.040

“*” uses Fisher exact probability method.

### 3.2 Multivariate logistic regression analysis

A binary logistic regression model was constructed, with moderately severe disease defined as the positive outcome. A forward–backward stepwise selection procedure was used for variable selection. Due to quasi-complete separation in the SIRS variable (94.3% of SIRS-positive patients developed moderately severe disease), which led to instability in conventional maximum likelihood estimation, the selected predictors (SIRS and 24-h TG level) were subsequently refitted using Firth’s penalized likelihood logistic regression to obtain more robust parameter estimates. The results indicated that SIRS remained the strongest independent risk factor (OR = 202.469, 95% CI: 33.662–1217.795, *p* < 0.001), while the 24-h TG level was also independently associated with disease severity (OR = 1.066, 95% CI:1.013–1.122, *p* = 0.013). Detailed results are presented in Table 4.

**Table 4:** Multivariable analysis using Firth’s penalized likelihood logistic regression.

Variables	*β* coefficient (standard error)	Wald χ^2^	*p* value	OR value (95%CI)
Intercept	−3.8172 (1.0574)	13.0318	0.0003	–
SIRS (Yes vs. Not)	5.3106 (0.9154)	33.6549	<0.0001	202.469 (33.663 ~ 1216.795)
24 h TG	0.0642 (0.0259)	6.1336	0.0133	1.066 (1.013 ~ 1.122)

### 3.3 Model validation results

**Figure 1:**
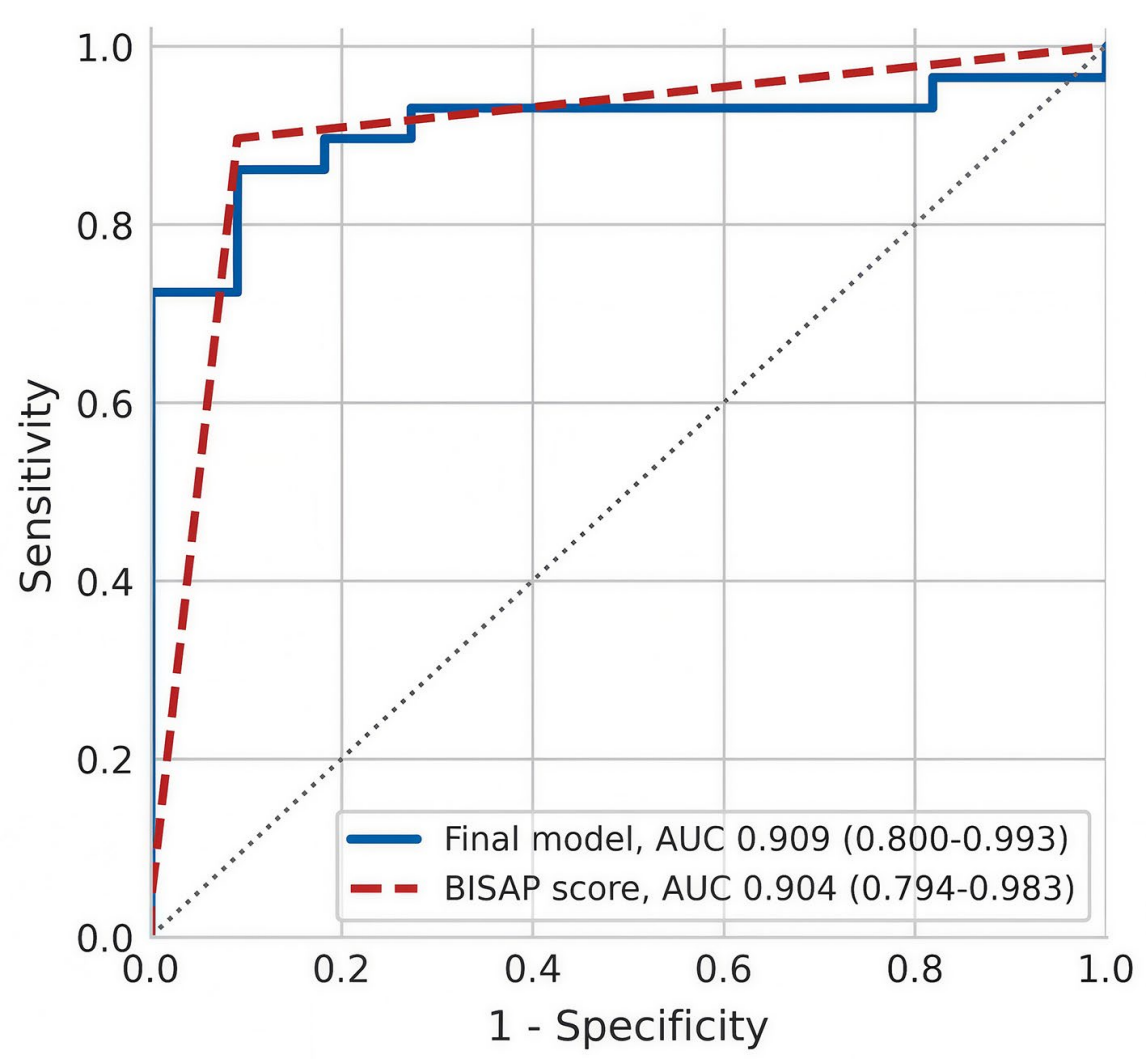
Comparison of ROC curves between the final Firth prediction model and the BISAP score in the validation set.

The final Firth penalized likelihood logistic regression model developed on the training set included two variables: SIRS and 24-h TG level. The probability of developing moderately severe disease was calculated using the following formula: *p* = 1/[1 + e^(−(−3.8172 + 5.3106 × SIRS + 0.0642 × 24hTG))]. After substituting each patient’s variables from the validation set (*n* = 40) into the model to obtain individualized predicted probabilities, the model achieved an AUC of 0.909 (95% CI: 0.804–0.990), with an optimal cutoff value of 0.865. The corresponding sensitivity, specificity, and accuracy were 86.2, 90.9, and 87.5%, respectively. The Brier score in the validation set was 0.115, and the calibration slope was 0.704. The Hosmer–Lemeshow test, performed after grouping by quartiles, yielded χ^2^ = 7.536 (*p* = 0.023). Given the small sample size of the validation cohort and the clustering of predicted probabilities at both extremes, the Hosmer–Lemeshow test is sensitive to grouping strategy and expected cell counts. Therefore, model performance was further evaluated using a comprehensive approach, incorporating the calibration curve, Brier score, calibration slope, DCA, and comparison with the BISAP score (Table 5; Figure 1).

**Table 5:** Discrimination and calibration performance of the final Firth prediction model in the training set, validation set, and overall data.

Dataset	MSAP *n*	AUC	95%CI Lower limit	95%CI Upper limit	Brier score	Calibration slope	Optimal cutoff value	Sensitivity	Specificity	Accuracy
Training (*n* = 106)	71	0.97	0.931	0.997	0.052	1.087	0.525	0.971	0.914	0.952
validation (*n* = 40)	29	0.909	0.804	0.99	0.115	0.704	0.865	0.862	0.909	0.875
Total (*n* = 146)	100	0.951	0.909	0.983	0.069	0.898	0.525	0.939	0.913	0.931

#### 3.3.1 The calibration curve, Brier score, calibration slope

**Figure 2:**
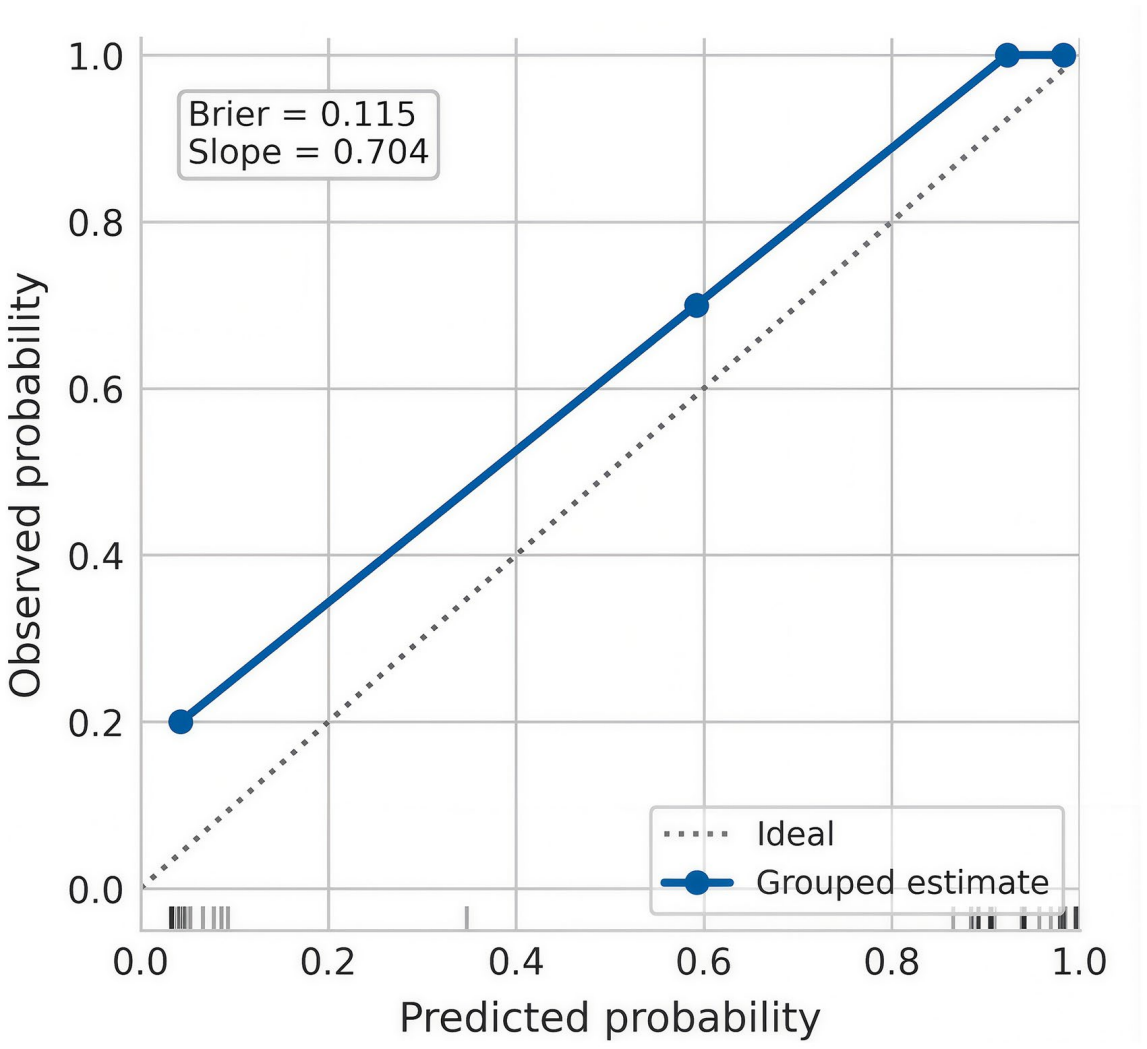
Calibration curve of the final Firth prediction model in the validation set.

The calibration curve demonstrated good overall agreement between predicted probabilities and observed event rates. However, in the low-risk subgroup, some deviation and underestimation were observed, likely due to the small sample size of the validation cohort and the clustering of predicted probabilities at the extreme ends. The Brier score in the validation set was 0.115, and the calibration slope was 0.704, suggesting an acceptable overall prediction error but indicating potential overfitting and slight overestimation of predictive strength. Further external validation with a larger sample size is warranted to confirm the model’s calibration performance (Figure 2).

#### 3.3.2 DCA

**Figure 3:**
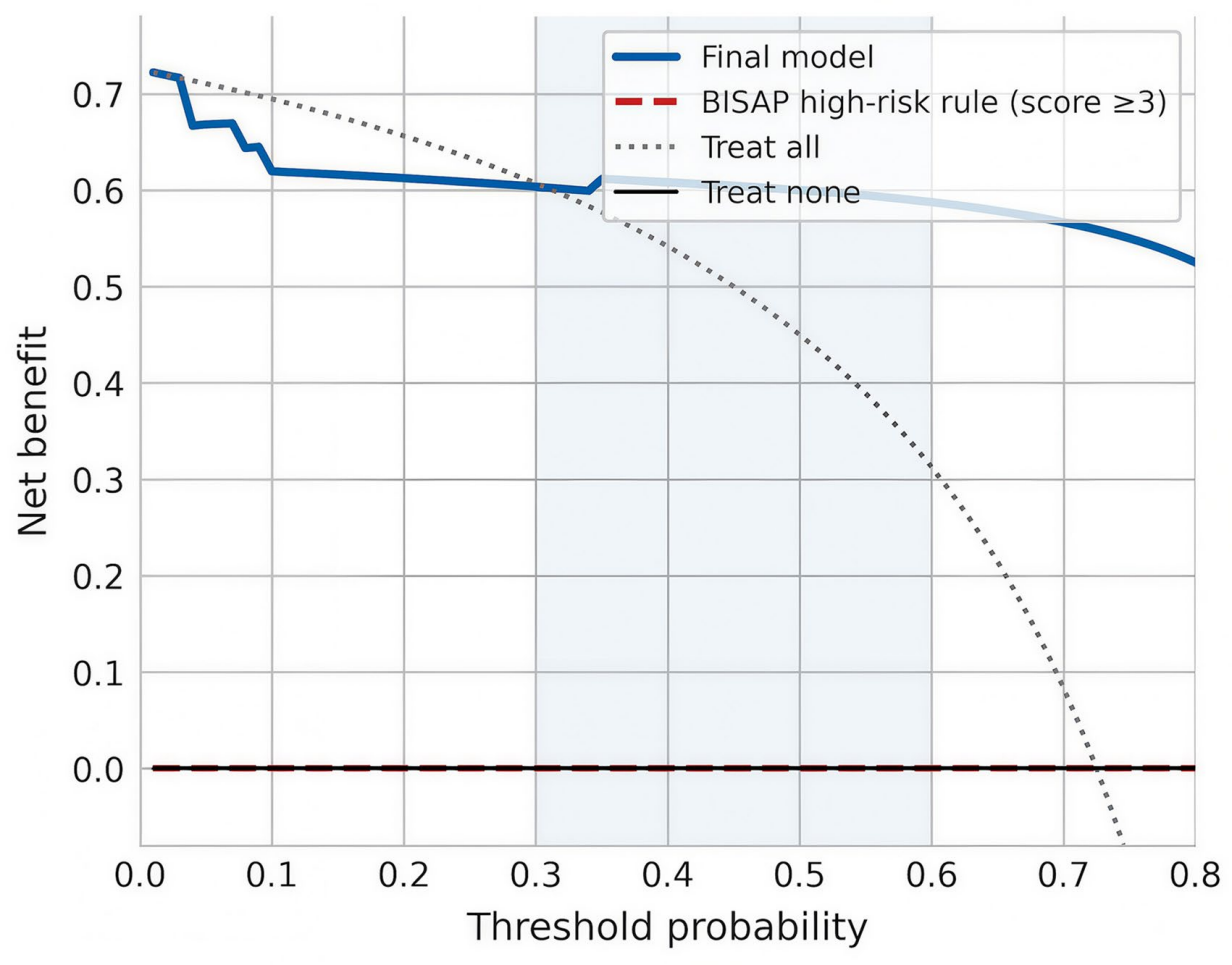
Decision curve analysis of the final Firth prediction model in the validation set.

To evaluate the potential clinical utility of the model, DCA was performed. The results showed that in the validation set, the net benefits of the final model at threshold probabilities of 0.40, 0.50, and 0.60 were 0.608, 0.600, and 0.588, respectively, which were higher than those of the treat-all strategy (0.542, 0.450, and 0.313, respectively), while the treat-none strategy remained at zero across all thresholds. These findings suggest that, within moderate to high threshold probability ranges where clinicians may opt for intensified monitoring or early intervention in patients at risk of moderately severe disease, the model provides greater net clinical benefit compared with either treating all or treating none strategies (Figure 3).

#### 3.3.3 Comparison with the BISAP score

In the validation set of this study, the 24-h BISAP score distribution was as follows: 13 patients scored 0, 26 scored 1, and 1 scored 2. No patient reached the conventional high-risk threshold of BISAP ≥3. Consequently, when using BISAP ≥3 to define high risk, the sensitivity was 0%, specificity was 100.0%, and accuracy was 27.5%. To avoid information loss associated with dichotomization in this cohort, ROC analysis was additionally performed using the original ordinal BISAP score. The final Firth-based model achieved an AUC of 0.909, while the BISAP score achieved an AUC of 0.904. The DeLong test indicated no statistically significant difference between the two models (*Z* = 0.105, *p* = 0.917). Detailed results are presented in Tables 6, 7 and Figure 1.

**Table 6:** Diagnostic performance of different BISAP cutoff values in the validation set.

BISAP cutoff value	Sensitivity	Specificity	Accuracy	TP	FP	TN	FN
≥1	0.897	0.909	0.9	26	1	10	3
≥2	0.034	1	0.3	1	0	11	28
≥3	0	1	0.275	0	0	11	29

**Table 7:** Comparison of AUCs between the final model and the BISAP score in the validation set (DeLong test).

Final model AUC	BISAP score AUC	AUC difference	*Z* value	*p* value
0.909	0.904	0.005	0.105	0.917

The above results suggest that the model developed in this study has discriminative performance comparable to that of the BISAP score. However, the proposed model provides individualized continuous predicted probabilities, offering greater granularity for risk stratification. In contrast, the conventional BISAP high-risk cutoff (≥3) failed to identify any high-risk patients in this cohort, which may be related to the distribution of age and BUN levels, as well as limitations in sample size.

#### 3.3.4 Bootstrap internal validation

To address the lack of true external validation, this study further performed internal validation using 1,000 bootstrap resampling iterations to estimate optimism in model performance. In the complete-case training dataset, the apparent AUC was 0.970, while the optimism-corrected AUC was 0.969. The optimism-corrected Brier score was 0.057, and the mean bootstrap calibration slope was 0.997. In the complete-case overall dataset, the apparent AUC was 0.951, the optimism-corrected AUC was 0.949, the optimism-corrected Brier score was 0.074, and the mean bootstrap calibration slope was 1.010. These results indicate minimal reduction in discriminative performance after optimism correction, suggesting good internal stability of the model. Detailed results are presented in Table 8.

**Table 8:** Bootstrap internal validation results.

Data set	MSAP *n*	Apparent AUC	Optimism-corrected AUC	Apparent Brier	Optimism-corrected Brier	Bootstrap calibration slope
Training (*n* = 106)	71	0.97	0.969	0.052	0.057	0.997
Total (*n* = 146)	100	0.951	0.949	0.069	0.074	1.01

### 3.4 Exploratory analysis of the predictive efficacy of 24 h TG alone

To further evaluate the clinical utility of 24-h TG as an independent risk indicator, ROC curve analysis was performed in the training set (*n* = 106). The results demonstrated that 24-h TG had moderate diagnostic value for distinguishing between mild and moderately severe disease, with an AUC of 0.690 (95% CI: 0.586–0.794, *p* = 0.002). The optimal cut-off value, determined using the maximum Youden index, was 18.41 mmol/L, corresponding to a sensitivity of 66.2%, a specificity of 65.7%, and a Youden index of 0.319.

This cut-off value (18.41 mmol/L) was then applied to the independent validation set (*n* = 40) for internal verification. Using this threshold, the sensitivity and specificity of 24-h TG for predicting moderately severe disease were 58.62% (95% CI: 40.70–76.54%) and 81.82% (95% CI: 59.03–100.00%), respectively. The PPV was 89.47%, the NPV was 42.86%, and the overall accuracy was 65.0% (95% CI: 50.22–79.78%).

In the validation set, this cut-off demonstrated higher specificity and PPV (increases of approximately 16 and 14 percentage points, respectively) but lower sensitivity (decrease of approximately 7 percentage points) compared with the training set results. This suggests that the cut-off is more reliable for ruling out patients at low risk of moderately severe disease, but it carries a modest risk of missed diagnoses. The observed difference in predictive performance may be partly attributable to the limited sample size of the validation cohort (Table 9).

**Table 9:** Diagnostic performance verification of 24 h TG critical value (18.41 mmol /L) in the validation set (*n* = 40).

Diagnostic index	Stats	95% Confidence interval	Training sets correspond to values
Sensitivity	58.62%	40.70–76.54%	66.20%
Specificity	81.82%	59.03–100.00%	65.70%
Positive predictive value	89.47%	-	-
Negative predictive value	42.86%	-	-
Accuracy rate	65.00%	50.22–79.78%	-

## 4 Discussion

HTG-AP is an important subtype of acute pancreatitis, characterized by rapid disease progression. However, few studies have focused on risk predictors capable of accurately identifying moderately severe patients within the first 48 h (8). Based on this retrospective study, we developed a simple model to predict progression to HTG-MSAP. The results demonstrated that SIRS and elevated TG levels within 24 h of admission were independent risk factors for progression to moderately severe disease. The prediction model incorporating these two variables performed well in the independent validation set, with an AUC of 0.909, sensitivity of 86.2%, specificity of 90.9% and accuracy of 87.5%. Compared with findings from previous studies (10, 11, 19, 20), our model showed superior predictive ability. Regarding calibration, the model showed a certain degree of miscalibration. Given the small sample size of the validation cohort and the clustering of predicted probabilities at the high and low extremes, the Hosmer–Lemeshow test is highly sensitive to grouping strategy and expected cell frequencies; therefore, its statistical significance may not accurately reflect true calibration performance. The calibration curve indicated that the overall relationship between predicted probabilities and observed incidence rates remained consistent. However, some underestimation and deviation were observed in the low-risk range, likely attributable to data sparsity within this risk interval due to the limited sample size. DCA further suggested that the model may provide clinical utility in decision-making, particularly for patients classified within the intermediate-to-high risk range.

The BISAP score is a severity assessment system for acute pancreatitis that evaluates patients using five readily available clinical and physiological indicators: age, pleural effusion, impaired mental status, blood urea nitrogen, and SIRS. It is characterized by simplicity, rapidity, and good specificity. Comparison between the model developed in this study and the BISAP score showed no statistically significant difference in AUC (DeLong test, *p* = 0.917), indicating comparable discriminative performance. However, the proposed model provides individualized continuous predicted probabilities, thereby avoiding the information loss associated with the dichotomization of risk using a fixed BISAP cutoff (≥3 points). In the validation cohort of this study, no patient reached a BISAP score ≥3, resulting in a sensitivity of 0% for the conventional high-risk threshold. In contrast, the proposed model was able to effectively identify patients with moderately severe disease, achieving a sensitivity of 86.2%. This suggests that in populations with relatively mild disease distribution or in specific subgroups, such as patients with lower age or lower BUN levels, continuous prediction models may offer greater adaptability than traditional categorical risk stratification tools.

To address the lack of external validation, we performed internal validation using 1,000 bootstrap resamples. The results showed that the apparent AUC of the training set was 0.970, which decreased slightly to 0.969 after optimism correction. Similarly, the apparent AUC of the overall dataset was 0.951, which decreased to 0.949 after correction, while the mean calibration slope remained close to 1. These findings suggest that both the discriminative ability and calibration performance of the model were minimally affected after optimism correction, indicating good internal stability and a low risk of overfitting. However, bootstrap-based internal validation cannot replace true external validation. Therefore, future studies are warranted to further assess the generalizability of the model using multicenter datasets, temporally distinct cohorts, or independent external validation cohorts.

Exploratory analysis also indicated that 24-h TG alone (cut-off: 18.41 mmol/L) had moderate predictive value (AUC = 0.690), but its performance was inferior to that of the combined model, particularly with respect to sensitivity. This suggests that TG alone is more reliable for ruling out low-risk patients, but carries a risk of missed diagnosis, highlighting the importance of using the combined model. This study identifies two key early warning indicators of HTG-AP progression within 24 h. SIRS can be assessed using vital signs and routine blood tests, while TG levels are readily measurable upon admission. Both indicators are easy to obtain, making them practical for very early risk stratification and providing clinicians with quantifiable markers to rapidly identify high-risk patients at the initial stage of hospitalization.

In both the training and validation sets, the incidence of SIRS in the moderately severe group was significantly higher than in the mild group. SIRS represents a systemic inflammatory response of the body to infectious or non-infectious injury. In acute pancreatitis, local inflammation resulting from pancreatic acinar cell injury can extend systemically, leading to SIRS, which may subsequently cause multiple organ failure or even death (21, 22). Previous studies have shown that the presence of SIRS on the first day of admission is a key predictor of SAP. Nearly all critically ill patients who develop persistent organ failure, pancreatic necrosis, require intensive care, or die exhibit SIRS on the first day of admission (23). Moreover, patients with SIRS persisting for more than 4 days during the first week of illness have a significantly higher incidence of infected pancreatic necrosis compared with those with shorter durations of SIRS (24). Early assessment of SIRS upon admission provides a rapid, simple, and powerful tool for identifying high-risk patients, enabling timely intensive monitoring and early therapeutic intervention. In this study, due to the presence of quasi-complete separation in the SIRS variable, we refitted the selected predictors (SIRS and 24-h TG level) using Firth’s penalized likelihood logistic regression. The results indicated that SIRS remained the strongest independent risk factor.

In the training set, the 24-h TG level of moderately severe patients was significantly higher than that of mild patients. When considered alone, a 24-h TG level above the critical value of 18.41 mmol/L was more reliable for ruling out patients at low risk of moderately severe disease. The pathogenesis of HTG-AP is not fully understood. One proposed mechanism is that excessive TG in the pancreas is hydrolyzed by pancreatic lipase, leading to the accumulation of free fatty acids, which damages acinar cells and pancreatic capillaries. This process creates ischemia and an acidic environment, further exacerbating the cytotoxicity of free fatty acids. Additionally, chylomicron-induced hypercoagulability in pancreatic capillaries may contribute to disease progression (25, 26). Several studies have reported that elevated TG levels are an independent risk factor for poor clinical outcomes, including pancreatic necrosis, organ failure, ICU admission, and death. Higher levels of TG are associated with worse outcomes, emphasizing the importance of routine TG monitoring and active intervention within 72 h of AP admission (27). Moreover, patients presenting with very high TG levels at admission (>2,648 mg/dL,29.9 mmol/L) have significantly higher rates of local complications and a greater likelihood of progression to moderately severe or severe pancreatitis (28). It has also been observed that when TG levels exceed 11.3 mmol/L, the risks of cardiac and renal organ failure, incidence of MSAP, and length of hospital stay increase significantly, whereas TG levels above 22.6 mmol/L are associated with a markedly higher risk of SAP (29). Collectively, these findings indicate that extremely high TG levels are not only a causative factor for HTG-AP but also serve as a predictor of disease severity and poor prognosis.

The results of this study indicated that, in the training set, the incidence of diabetes mellitus, diabetic ketosis, and diabetic ketoacidosis was significantly higher in the moderately severe group than in the mild group, with all differences reaching statistical significance. AP damages the pancreas through the activation of inflammatory mediators and impairs insulin action in peripheral tissues. Patients with pre-existing diabetes already have defects in insulin secretion or utilization. When complicated by ketoacidosis, pancreatic inflammation can further impair islet function and reduce insulin secretion, substantially increasing the risk of hyperglycemia and metabolic complications (30). Previous studies have demonstrated that diabetes is an important determinant of poor clinical outcomes in MSAP and SAP patients, particularly with respect to local complications (31). These findings underscore the necessity of early and intensive intervention for HTG-AP patients with a history of diabetes.

Univariate analysis revealed multiple dynamic changes in laboratory parameters related to disease severity; however, none demonstrated predictive value within the first 24 h. In the training set, patients in the moderately severe group had significantly higher WBC and CRP levels at 72 h, while HCT, HGB, and Ca^2+^ levels were significantly lower compared with the mild group. Similar trends of increased WBC and CRP and decreased Ca^2+^ at 72 h were also partially observed in the validation set. CRP levels greater than 150 mg/L within 48 h of admission are widely recognized as indicative of severe pancreatitis. CRP, a positive acute-phase reactant synthesized by the liver, is commonly used in the diagnosis, prognosis, and mortality prediction of inflammatory diseases (32). Early studies have confirmed that elevated CRP levels can predict the severity of AP (33), and numerous studies have associated high CRP levels with severe AP progression and pancreatic necrosis (12). Although CRP is a highly applicable clinical inflammation marker, it peaks relatively late (48–72 h) and may not directly reflect infection status (34). Ca^2+^ is an easily measurable biochemical parameter in clinical practice. Hypocalcemia is a classic predictor of poor prognosis in AP, potentially due to the role of Ca^2+^ ions in pancreatic cell apoptosis and inflammatory responses. Previous studies have demonstrated that early Ca^2+^ reduction is an independent predictor of severe HTG-AP and is closely associated with poor outcomes (35). Consistently, in this study, Ca^2+^ levels at 72 h were lower in the moderately severe group compared with the mild group. While not statistically significant as an early predictor, dynamic monitoring of Ca^2+^ remains clinically important for prognostic assessment. HCT, defined as the fraction of red blood cells in anticoagulated whole blood after centrifugation, reflects both inflammatory response and oxygen delivery capacity, as well as blood concentration and potential bleeding or anemia. Hypovolemia, third-space fluid loss, and pancreatic necrosis are common in AP pathophysiology, making HCT a useful indicator of these processes (36). Elevated HCT increases blood viscosity, reduces tissue perfusion, and can induce hypoxia and organ dysfunction; it may also promote microcirculatory disorders, red blood cell aggregation, and microthrombosis, exacerbating regional ischemia and inflammation (37). Conversely, low HCT can also be associated with poor prognosis, likely due to anemia or hemodilution, which compromise tissue oxygen delivery and systemic metabolic stability (38, 39). These findings align with the present study, in which HCT and HGB at 72 h were lower in the moderately severe group than in the mild group. Monitoring HCT thus provides valuable insight into the effects of early fluid resuscitation, circulatory status, and supports individualized treatment decisions.

These dynamic laboratory indicators confirm that HTG-MSAP is associated with persistent inflammation and an increased risk of local complications. Although they are not practical as early predictors, they can serve as valuable auxiliary markers for disease monitoring and treatment evaluation. Specifically, the 24-h TG level, in combination with the predictive model, can be used for early risk stratification, while CRP, Ca^2+^, HCT, and other laboratory parameters at 72 h can help assess disease progression and therapeutic response. The complementary predictive value of markers at different time points allows for a more comprehensive evaluation of the disease course and underscores the clinical significance of whole-process management in HTG-AP.

This study has several limitations. First, as a single-center retrospective cohort study, the overall sample size was relatively small, particularly in the internal validation set, which may affect the stability of the statistical results and the accuracy of the estimated performance metrics of the model. Although we performed 1,000 bootstrap resamples for internal validation, which demonstrated good model stability, this approach cannot replace external validation using data from different centers or distinct time periods. Second, the current predictive model includes only two early variables. While this enhances clinical applicability and ease of use, it may not fully capture additional dynamic clinical or laboratory indicators that could further improve predictive performance. Third, we excluded patients with a hospital stay of less than 72 h, which may introduce selection bias. This exclusion criterion was primarily based on the primary outcome definition according to the 2012 Revised Atlanta Classification, which requires a complete assessment within 72 h of disease onset. Patients with very short hospital stays often present with mild disease and are discharged early; therefore, their exclusion ensured that all included patients had complete 72-h clinical observation data and helped avoid outcome misclassification due to incomplete information. Nevertheless, this criterion may have systematically excluded milder cases, potentially resulting in a cohort with relatively higher disease severity and possibly overestimating the apparent performance of the predictive model. Fourth, future studies should extend the observation period and incorporate in-hospital mortality and various complications as secondary outcomes to more comprehensively assess the prognostic value of the model.

## 5 Conclusion

SIRS and elevated TG levels within 24 h of admission are independent risk factors for MSAP in HTG-AP, and the predictive model demonstrates good performance. A 24-h TG level below 18.41 mmol/L suggests a low risk of progression to moderately severe disease. These findings also highlight the importance of using a combined model incorporating multiple biochemical indicators for early risk prediction, although the model’s effectiveness requires further validation in larger and external cohorts.

## Data Availability

The original contributions presented in the study are included in the article/supplementary material, further inquiries can be directed to the corresponding author.
